# Enhancing Cancer care of rural dwellers through telehealth and engagement (ENCORE): protocol to evaluate effectiveness of a multi-level telehealth-based intervention to improve rural cancer care delivery

**DOI:** 10.1186/s12885-021-08949-4

**Published:** 2021-11-23

**Authors:** Tuya Pal, Pamela C. Hull, Tatsuki Koyama, Phillip Lammers, Denise Martinez, Jacob McArthy, Emma Schremp, Ann Tezak, Anne Washburn, Jennifer G. Whisenant, Debra L. Friedman

**Affiliations:** 1 Vanderbilt-Ingram Cancer Center, 2220 Pierce Avenue, Nashville, TN 37232 USA; 2 Division of Genetic Medicine, Department of Medicine, Vanderbilt University Medical Center, 2220 Pierce Avenue, 536 Robinson Research Building, Nashville, TN 37232 USA; 3 University of Kentucky Markey Cancer Center, 800 Rose Street, Lexington, KY 40536 USA; 4 Department of Behavioral Science, University of Kentucky College of Medicine, Rural and Underserved Health Research Center, Healthy Kentucky Research Building, 760 Press Avenue, Lexington, KY 40536 USA; 5 Department of Biostatistics, Vanderbilt University Medical Center, 2525 West End, Suite 1100, Nashville, TN 37203 USA; 6 Baptist Cancer Center, 80 Humphreys Center Suite 330, Memphis, TN 38120 USA; 7 Division of Pediatric Hematology & Oncology, Department of Pediatrics, Vanderbilt University Medical Center, 395 Preston Research Building, 2220 Pierce Avenue, Nashville, TN 37232 USA; 8 Division of Hematology & Oncology, Department of Medicine, Vanderbilt University Medical Center, 1161 21st Avenue South, Nashville, TN 37232 USA

**Keywords:** Virtual molecular tumor board, Consolidated framework for implementation research (CFIR), Precision medicine

## Abstract

**Background:**

Despite lower cancer incidence rates, cancer mortality is higher among rural compared to urban dwellers. Patient, provider, and institutional level factors contribute to these disparities. The overarching objective of this study is to leverage the multidisciplinary, multispecialty oncology team from an academic cancer center in order to provide comprehensive cancer care at both the patient and provider levels in rural healthcare centers. Our specific aims are to: 1) evaluate the clinical effectiveness of a multi-level telehealth-based intervention consisting of provider access to molecular tumor board expertise along with patient access to a supportive care intervention to improve cancer care delivery; and 2) identify the facilitators and barriers to future larger scale dissemination and implementation of the multi-level intervention.

**Methods:**

Coordinated by a National Cancer Institute-designated comprehensive cancer center, this study will include providers and patients across several clinics in two large healthcare systems serving rural communities. Using a telehealth-based molecular tumor board, sequencing results are reviewed, predictive and prognostic markers are discussed, and treatment plans are formulated between expert oncologists and rural providers. Simultaneously, the rural patients will be randomized to receive an evidence-based 6-week self-management supportive care program, Cancer Thriving and Surviving, versus an education attention control. Primary outcomes will be provider uptake of the molecular tumor board recommendation and patient treatment adherence. A mixed methods approach guided by the Consolidated Framework for Implementation Research that combines qualitative key informant interviews and quantitative surveys will be collected from both the patient and provider in order to identify facilitators and barriers to implementing the multi-level intervention.

**Discussion:**

The proposed study will leverage information technology-enabled, team-based care delivery models in order to deliver comprehensive, coordinated, and high-quality cancer care to rural and/or underserved populations. Simultaneous attention to institutional, provider, and patient level barriers to quality care will afford the opportunity for us to broadly share oncology expertise and develop dissemination and implementation strategies that will enhance the cancer care delivered to patients residing within underserved rural communities.

**Trial registration:**

Clinicaltrials.gov, NCT04758338. Registered 17 February 2021 – Retrospectively registered, http://www.clinicaltrials.gov/

**Supplementary Information:**

The online version contains supplementary material available at 10.1186/s12885-021-08949-4.

## Background

### Rationale

Several studies have documented elevated cancer incidence and/or mortality in rural compared to urban communities [1–7], particularly pronounced in certain regions of the United States (US), including the South [8]. These differences have broad population health implications as almost 20% of Americans live in rural areas [8, 9], but only 10% of physicians practice there [10]. Rural counties have some of the highest rates of poverty, where residents are faced with limited availability to cancer treatment and supportive care services, transportation barriers, and financial issues [11–15]. Similarly, challenges are faced by rural providers in delivering cancer care to their patients, with limited access to comprehensive care being the most important reason for outcome disparities [16]. Moreover, the growing divide in mortality between rural and urban dwellers [1, 5, 17–19] highlights the need to evaluate multi-level interventions directed at providers and their patients to improve outcomes.

Suggested strategies to enhance rural cancer care to alleviate issues associated with long travel distances comprise technology-based approaches to deliver remote care delivery, inclusive of education and supportive care [12]. Telehealth use in rural communities allows extension of care to wide geographic regions without patient or provider travel. An Institute of Medicine report noted that telehealth for rural hospitals drives volume and increases quality of care, while preserving low cost [20]. Telehealth has been successfully used for cancer screening, treatment, and supportive care [21–26], and been expanded even further especially in the post COVID-19 era [27–31].

Provision of optimal cancer care delivery also requires multispecialty and multidisciplinary input. Tumor boards contribute to such input and provide evidence- and consensus-based data to optimize cancer treatment and can be accessed virtually with interactive participation from large geographical regions [32–34]. With the advent of next generation sequencing (NGS), the use of tumor boards has expanded to meet new challenges faced for physicians and patients, with respect to interpretation of results and translation to clinical interventions. To meet this need, we and others have developed successful telehealth-based molecular tumor boards, which enable community oncologists to present cases in real time to an academic cancer center [35–37]. This is particularly salient in rural communities, where there are fewer oncologists and, subsequently, are faced with the challenge of being well versed in the rapidly-changing molecular architecture of multiple tumor types. Additionally, late stage disease at initial diagnosis may be more common in rural patients [4] where molecular tumor diagnostics are more likely to direct targeted treatments. Therefore, telehealth-based molecular tumor boards offer a mechanism to disseminate evidence-based information from a National Cancer Institute (NCI)-comprehensive cancer center to its rural catchment area.

### Supportive care self-management interventions

Self-management support is the systematic provision of education and supportive care to increase the patients’ skills and confidence in managing their health problems and is an integral component of the Chronic Care Model [38–42]. Self-management interventions for cancer have been developed with face-to-face, online and telehealth models to minimize treatment-related symptoms and distress, improve treatment adherence and psychosocial wellbeing, and promote survival [39, 43–48]. The Chronic Disease Self-Management Program (CDSMP) is an evidence-based self-management intervention with demonstrated efficacy across numerous chronic health conditions with dissemination across the US, inclusive of rural communities, and to 25 countries [40, 49–53]. The CDSMP adaptation for cancer patients, Cancer Thriving and Surviving (CTS), has demonstrated efficacy in improving patient-provider communication, energy, and sleep, and reducing depression and stress-related problems [48, 54]. Yet access to such programs is limited in rural communities with wide geographic areas. Therefore, evaluation of the effectiveness of the evidence-based CTS intervention delivered through telehealth among rural patients is needed given its potential to improve patient outcomes.

### Objectives and aims

**Fig. 1:**
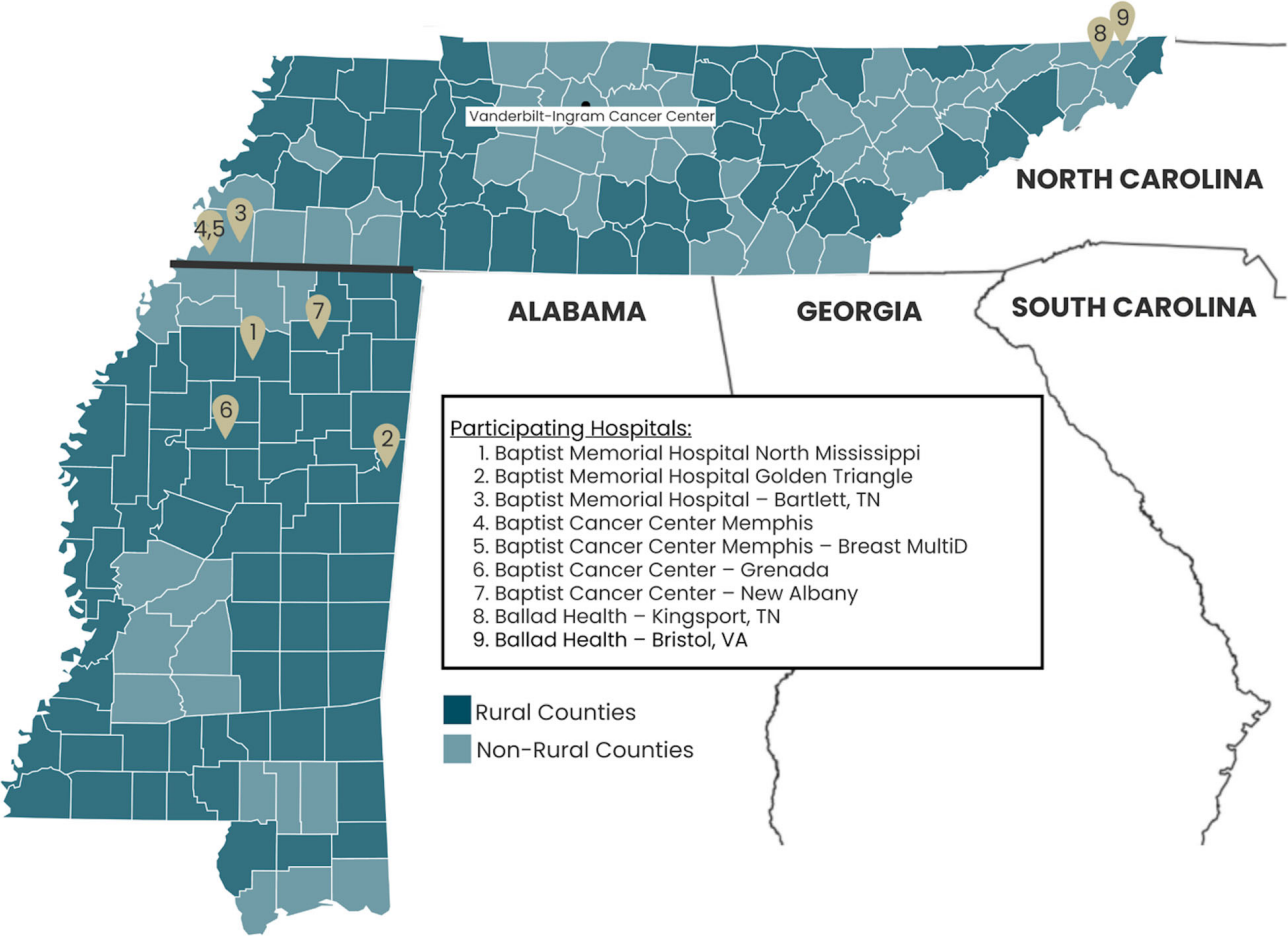
Location of Participating Vanderbilt Health Affiliate Network sites serving rural counties within the VICC Catchment area. This figure illustrates the study’s catchment area throughout Tennessee and Mississippi, including a distinction between rural versus non-rural counties. Each number represents the location of one of nine participating hospitals through the Vanderbilt Health Affiliated Network (VHAN), including seven through the Baptist Memorial Health Care Corporation and two through Ballad Health. This figure was created by the authors specifically for this clinical trial protocol

The goal of this project is to assess improvement in delivery of quality cancer care using a multi-level telehealth technology intervention tested at both the provider level and the patient level in the rural catchment area of Vanderbilt-Ingram Cancer Center (VICC), an NCI-designated Comprehensive Cancer Center (Fig. 1). To achieve this goal, this study has two specific aims:

#### Aim 1

Evaluate the clinical effectiveness of a multi-level telehealth-based intervention for rural hospitals consisting of provider access to tumor board expertise that incorporates disease, patient, and molecular tumor characteristics, together with patient access to a supportive care intervention to improve cancer care delivery.

#### Aim 2

Identify the facilitators and barriers to future larger scale dissemination and implementation of the multi-level intervention, designed to enhance the quality of rural cancer care delivery.

### Conceptual framework for implementation

**Fig. 2:**
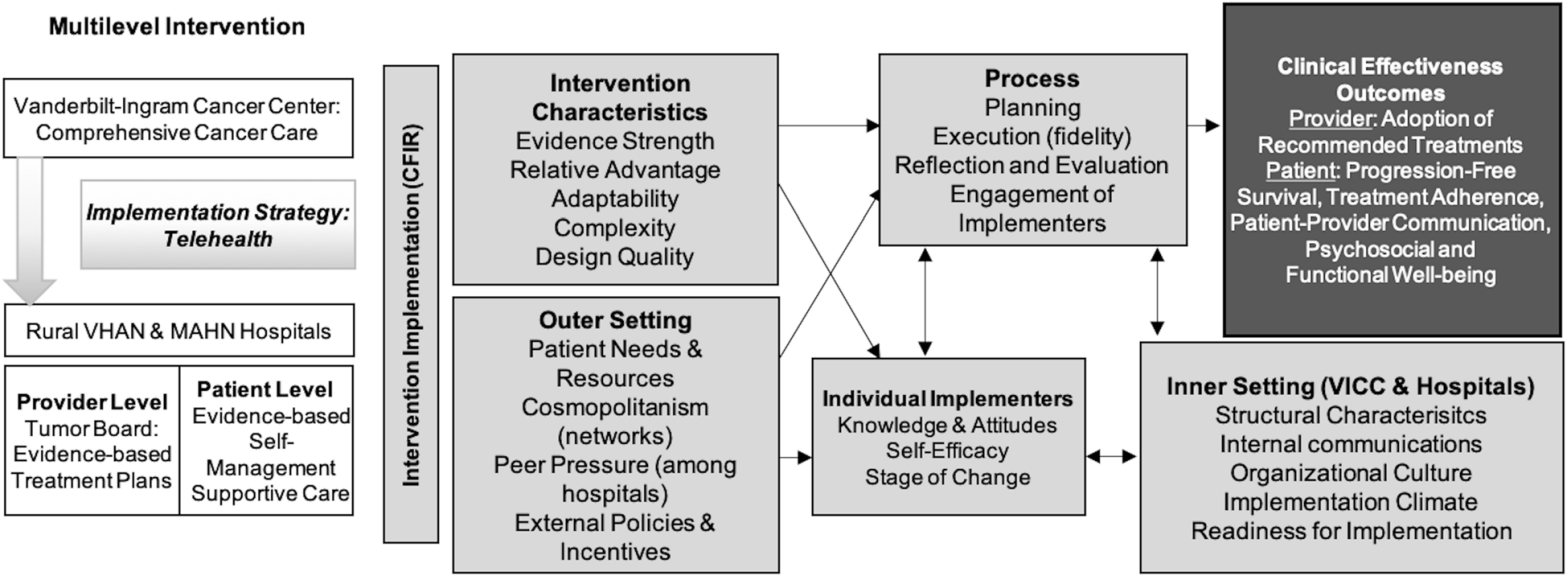
Conceptual Framework: Implementation of Evidence-Based Cancer Care Delivery to Rural Populations via Telehealth. This figure illustrates the conceptual framework for implementation of the multi-level telehealth intervention to bring evidence-based comprehensive cancer care to patients in rural counties in Tennessee. The white blocks on the left indicate that we will evaluate the clinical effectiveness of delivering a multi-level telehealth intervention to rural hospitals to improve cancer care delivery, consisting of 1) provider-level access to tumor board expertise that incorporates disease, patient, and molecular tumor characteristics, and 2) patient-level access to a supportive care intervention (Aim 1). The dark gray block on the top right outlines the intervention outcomes. The gray blocks in the middle illustrate domains and subdomains of the CFIR that we will examine during intervention delivery to identify barriers and facilitators to future larger scale dissemination and implementation of the intervention (Aim 2)

In order to identify potential barriers and facilitators that may influence implementation and future dissemination and to understand “what ‘works where and why’ across multiple contexts” during and after implementation [55, 56], an implementation framework is helpful. The Consolidated Framework for Implementation Research (CFIR), a meta-theoretical framework that delineates a taxonomy of concepts to examine the process of implementation [55], was chosen for the current study as outlined in Fig. 2. The CFIR Outer Setting domain includes factors outside of the implementing organizations such as: patient needs and resources, the degree to which the hospitals are networked with external organizations (cosmopolitanism), peer competition with other hospitals, and external policies and incentives, such as evidence-based expert practice guidelines and criteria for treatment and reimbursement. The Inner Setting domain reflects characteristics of the organizations implementing the intervention (VICC and rural hospitals): structural characteristics (organization size, age, maturity), the nature and quality of organization’s internal communications, general organizational culture, implementation climate (intention for change, compatibility, relative priority, organizational incentives, goals/feedback, learning climate), and the organization’s interventional readiness (leadership engagement, available resources for sustainability, access to information).

## Methods/design

### Overall study design

**Fig. 3:**
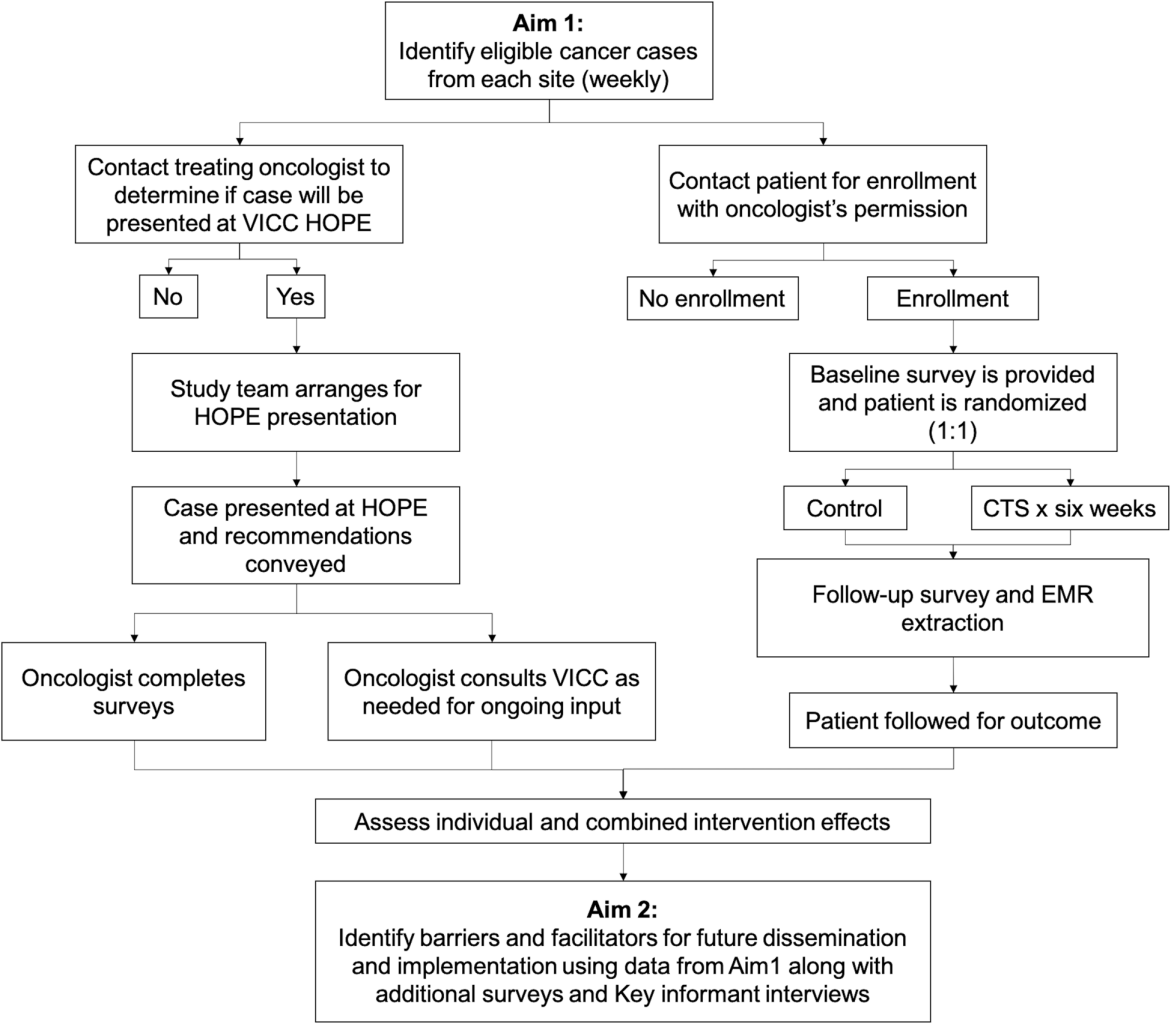
Study schema. VICC, Vanderbilt-Ingram Cancer Center; HOPE, Hereditary and Oncologic Personalized Evaluation; CTS, Cancer Thriving and Surviving intervention; EMR, electronic medical record

This study will utilize information technology (IT)-enabled, team-based care delivery models to provide comprehensive, coordinated, high-quality cancer-related care focused on rural populations. The study schema is presented in Fig. 3. We will evaluate the clinical effectiveness of a multi-level telehealth-based intervention in hospitals that see large volumes of rural patients. The intervention consists of provider access to tumor board expertise that incorporates disease, patient, and molecular tumor characteristics along with patient access to a supportive care intervention to improve cancer care delivery (Aim 1), and identification of facilitators and barriers to enable future larger scale dissemination and implementation of the multi-level intervention that is designed to enhance the quality of rural cancer care delivery (Aim 2). Simultaneous attention to institutional, provider, and patient level barriers to quality care will provide the opportunity for VICC, located in a region of the country with some of the highest cancer mortality rates [57], to broadly share oncology expertise, as well as develop dissemination and implementation strategies to remotely enhance patient care to underserved rural communities.

### Study setting

VICC is the sole NCI-designated Comprehensive Cancer Center in Tennessee serving adult as well as pediatric cancer patients. The geographic placement of VICC, as well as the regionally-renowned expertise in cancer care, presents a tremendous opportunity to develop strategies to address regional disparities in rural cancer care, including remote access to oncology expertise and supportive care.

Participating healthcare sites will include multiple oncology practices (i.e., cancer clinics) within the Vanderbilt Health Affiliated Network (VHAN), including the Baptist Memorial Health Care Corporation and Ballad Health. Participants will include oncology providers and patients at the aforementioned participating healthcare sites. The eligibility criteria are: 1) At least 21 years of age or older and English speaking with the ability to provide informed consent; 2) Oncology providers must treat patients within the designated VHAN oncology practices; 3) Patients with newly diagnosed or relapsed disease that are patients of the aforementioned oncology providers; and 4) Access to a reliable, video-capable device with internet access.

### Data collection

**Fig. 4:**
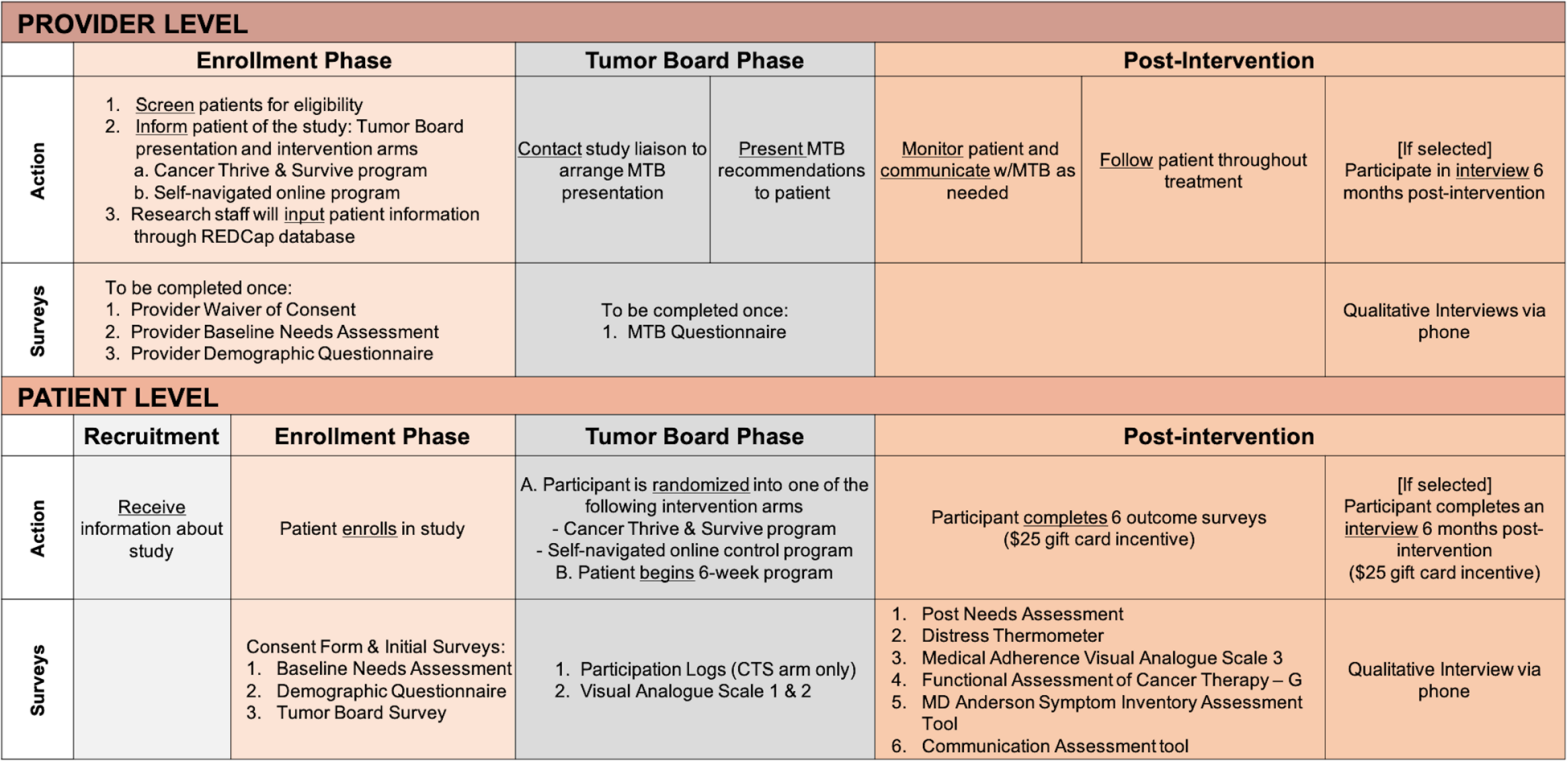
Recruitment and surveys conducted at the provider level and patient level per study phase. The action and particular survey that will be completed by both the provider and patient for the Enrollment Phase, Tumor Board Phase, and Post-Intervention Phase is illustrated. MTB, molecular tumor board; CTS, Cancer Thriving and Surviving

This study will implement provider-level and patient-level interventions using a mixed-methods approach. The CFIR will be used to examine intervention delivery to identify barriers and facilitators to future dissemination and implementation of the intervention [55, 56]. Both quantitative and qualitative data collection measures will be used, including baseline surveys before intervention implementation surveys, qualitative interviews, and follow-up surveys after intervention. Provider and patient recruitment along with the various surveys that will be conducted per study phase (i.e., Enrollment Phase, Tumor Board Phase, and Post-Intervention Phase) are presented in Fig. 4.

#### Provider-level intervention and data collection

The provider will be asked to complete a baseline survey to collect demographic information, including gender, age, race, ethnicity, and number of years post fellowship training. In addition to demographics, this survey will also assess the financial, emotional, and educational needs of rural cancer patients, and interest in expansion of telehealth services. To measure relative advantage (CFIR intervention characteristic), providers will be asked to rate on a scale of 1–10 the perceived need for remote access to the molecular tumor board, perceived importance of educational and self-management interventions for their patients, and interest in participating in future research. An open-ended qualitative item will ask for suggestions of needed support for the intervention.

At the tumor board, genomic, histologic, and clinical data will be presented for further discussion and recommendations. The oncologist, with their patient, will decide if they will proceed with the recommendations, or alternatively, start another treatment. Recommendations and treatment changes will be collected.

Key informant interviews will be conducted with up to 10 participating oncologists at two points in time: 1) early implementation phase (six months after intervention initiation); and 2) maintenance phase (two years after initiation). The interview questions are adapted from the existing CFIR Interview Guide [58], covering the selected constructs under each of the five CFIR major domain. Interviews will be conducted by telephone using a semi-structured interview guide and will last about 20 min; all interviews will be audio recorded.

#### Patient-level intervention and data collection

If the patient expresses interest in the study, the provider will confirm eligibility then prompt them to complete the informed consent. If reliable access to a device with video and internet access is not available, a device will be mailed to patient’s home after they enroll and a data plan will be provided. These two resources will come at no cost to the patient, and the device will be returned at the conclusion of the study. Reasons for lack of participation will be tracked as a potential barrier to future dissemination.

Consented participants will be asked to complete a baseline survey to collect demographic information, including race, ethnicity, highest level of education, annual household income, and zip code; age and gender will be extracted from the medical record. Participants will then be randomized (1:1) into the CTS Program Arm or Education Attention Control Arm (Fig. 3). Participants randomized to CTS will have a 6-week intervention delivered via telehealth using the VUMC telehealth services (Table 1).

**Table 1:** Cancer Surviving and Thriving Syllabus

Week 1: • Introduction to the Workshop • Group Introductions • The Mind/Body Connection/Distraction • Fatigue Management and Getting Help • Introduction to Action Plans	Week 4: • Feedback • Cancer and Body Changes • Health Eating • Communication Skills • Problem-Solving • Making an Action Plan
Week 2: • Feedback/Problem Solving • Dealing with Difficult Emotions • Getting a Good Night’s Sleep • Regaining Fitness During and after Cancer Treatment • Making an Action Plan	Week 5: • Feedback • Endurance Exercise • Making Decisions about Treatment and Complementary Therapies • Maintaining a Healthy Weight • Dealing with Depression • Positive Thinking • Making an Action Plan
Week 3: • Feedback • Managing Plan • Living with Uncertainty • Making Decisions • Future Plans for Healthcare • Making an Action Plan	Week 6: • Feedback/Problem-Solving • Cancer and Relationships • Guided Imagery • Working with Your Healthcare Professional • Looking Back and Planning for the Future

Key informant interviews will be conducted with up to 25–30 patient participants in the CTS arm of the study approximately 6 months after the last session of the intervention. An index combining all of the patient-level outcome variables at pre-intervention and post-intervention will be created to calculate the pre-post difference with positive values indicating an improvement and negative values indicating a decrease. The interview questions are adapted from the existing CFIR Interview Guide [58], covering the selected constructs under each of the five CFIR major domain. Interviews will be conducted by telephone using a semi-structured interview guide and lasting about 20 min; all interviews will be audio recorded.

Participants in the Education Attention Control Arm will receive publicly available online educational materials compiled in a password protected website that broadly covers the same topics as the CTS syllabus and enables the study team to track how the participant navigates through these educational materials. All materials are drawn from preexisting public domains such as the National Institutes of Health, NCI, and the American Society of Clinical Oncology.

### Data analysis

Data will be analyzed sequentially in three phases: quantitative-qualitative-quantitative (i.e., baseline survey, interviews, and follow-up surveys). We will use connecting processes to combine both types of data, thus allowing one dataset to build upon another dataset in each sequential phase.

#### Process evaluation data

Implementation penetration will be assessed as a ratio of number of patients who participate in each group divided by total number of potential participants at each recruitment site. The recruitment rate will be calculated as the number of weeks required to recruit at least eight participants in each arm of the patient-level component. To address execution, three dimensions of intervention fidelity for the CTS program will be measured. Adherence of the group facilitators to the CTS protocol will be measured using an adapted Fidelity Checklist [59] completed by the facilitators to document completion of required activities. Exposure of patients to the intervention will be measured using participation/attendance logs and website analytics on usage of the online information. Participant responsiveness will be measured as satisfaction with the intervention from the post surveys. These measures will be combined into a fidelity score. Other data collected will include patient cancer type and stage, institution size, practice size, and number of rural counties with persistent poverty served and deprivation index, as indicators of structural characteristics.

#### Qualitative data

Audio recordings will be transcribed with no identifiers. Qualitative software (e.g., ATLAS.ti or RQDA) will be used to code the transcripts using the CFIR Codebook Template [58]. A thematic analysis method to identify and analyze commonly recurring themes will be used [60], then themes will be summarized for each group. Open-ended qualitative responses from surveys will be coded into categories based on emergent themes, then merged with the quantitative data sets as categorical variables.

#### Quantitative data and intervention outcomes

Survey data will primarily be summarized with descriptive statistics (i.e., frequencies/percentages for categorical variables and means/standard deviations for continuous variables) along with bivariate comparisons across sites and between provider and patient data, where appropriate using chi-squared tests, t-tests, and analysis of variance.

The primary outcomes will be provider uptake of the VICC HOPE molecular tumor board treatment recommendation and patient treatment adherence. Secondary outcomes include progression-free survival (PFS), patient functional and psychosocial well-being, and patient-provider communication. Outcomes will be compared between the CTS and attention control groups. Table 2 provides a list of the tools that will be utilized to measure these outcomes.

**Table 2:** Assessment surveys that will be used to evaluate outcomes from the provider and patient

Name of Tool	Measurement
The Visual Analogue Scale [61]	Patients mark a line at the point along a continuum showing how much of each drug they have taken in the past month
The Distress Thermometer [62]	Level of patient distress (0–10 scale) and problems contributing to it
The Functional Assessment of Cancer Therapy [63]	Health related quality of life in four primary domains: physical well-being, social/family well-being, emotional well-being, and functional well-being
The MD Anderson Symptom Inventory [64]	Thirteen common symptoms faced by cancer patients for severity and interference with aspects of daily life on a 10-point numeric rating scale
The Communication Assessment Tool [65]	Patients rate communication with the physician on a 15-item instrument that employs a five-point response scale

For the provider primary outcome, a logistic regression model will be fit with the following patient-level, provider-level, and institution-level variables as potential effect modifiers. Patient-level includes: cancer type, race, ethnicity, age and gender; provider-level includes: age, years since completion of fellowship training, race, ethnicity, gender; and institution-level includes: institution size (number of inpatient beds and outpatient infusion chairs), practice size (oncologists, nurses, advanced practice providers) and number of rural counties with persistent poverty served. Note that the outcome will most likely be correlated within the same provider, and to account for this correlation, we will use generalized estimating equations (GEE) with an independent weight matrix. Because the CTS Program Arm will be delivered to a group of 8 to 10 patients at a time, we will consider this as a crossed random effect. The Education Attention Control Arm will not have groups, and we will treat each individual as their own group to fit the model. Institution-level data will be used as fixed effects.

The secondary endpoints will be analyzed similarly using the regression approach taking into account the potential clustering effect of the treatment delivery group and practice. For a continuous outcome, mixed-effects model will be used; for PFS, we will use mixed effects Cox regression model to account for the intervention group clusters.

#### Sample size and power analysis

We expect to present 450 cases to the molecular tumor board, and we expect 300 patients to be randomized at 1 to 1 to the two treatment groups. For the patient-level intervention, we can detect treatment effect (difference in binary outcome) if the true successful (adherence) proportions are 75% (telehealth) and 60% (control) with 80% power while controlling type I error rate at 5%.

## Discussion

Participation in this study will directly test the clinical effectiveness of a telehealth implementation strategy for disseminating genomic cancer information for optimal patient care across diverse communities within and beyond the catchment area of VICC. This will allow iterative development of best practices for cancer care delivery to rural communities. This study is testing a novel multi-level intervention with simultaneous evaluation of provider and patient components to improve cancer care among underserved individuals in rural communities. A multi-level intervention to assess improvement in delivery of quality cancer care will be tested: 1) at the provider level through access to an existing telehealth-based molecular tumor board and availability to disease-specific expertise at VICC; and 2) the patient level through a telehealth-based supportive care intervention.

Using the CFIR model, we chose a multi-level intervention that includes both provider and patient level components. However, as such, it may be difficult to determine the relative contribution of each of the components. To overcome this limitation, we have developed some analyses of each component for Aim 1 and will look across institutional, provider, and patient levels for barriers and facilitators for future larger scale implementation in Aim 2. In addition, there may be cases with only an oncologist or patient participant. While we don’t expect this to be a high number, discordant participation will reflect real world circumstances and inform future dissemination and implementation strategies.

As with all telehealth services, this study could be challenged by technical difficulties. However, VICC has successfully brought telehealth into rural counties with low broadband connectivity. This includes combined use of computers, tablets and smart phones for the participants. To date, we have had no issues with VICC telehealth across a number of service lines and research projects. Additionally, through the Vanderbilt University Medical Center (VUMC), an institutional infrastructure exists for healthcare providers to deliver telehealth-based services. This existing VUMC telehealth infrastructure and software is available for use in the proposed study and access to broadband and devices will be provided to participants if needed. This will inform future scaling of this intervention.

### Summary and impact

With the proposed multi-level remote intervention, we will 1) improve comprehensive cancer care delivery to patients residing in rural communities with persistent poverty; 2) use telehealth to broaden the reach of our NCI-designated comprehensive cancer center in these communities; 3) disseminate evidence-based information to rural oncology providers using a tumor board to share expertise in molecular tumor analysis and interpretation; 4) provide guidance on cutting-edge therapies based on patient, disease, and tumor characteristics; and 5) offer an evidence-based self-management intervention to rural patients. Furthermore, we will collect data for future dissemination and implementation of this intervention in order to decrease disparities in rural cancer patients with limited access to comprehensive care.

## Supplementary Information

**Additional file 1.**

## Data Availability

Not applicable. Sharing of data generated from this study will adhere to the National Institutes of Health guidelines regarding sharing of data collected using federal funds.

## Abbreviations

CDSMP: Chronic Disease Self-Management Program
CFIR: Consolidated Framework for Implementation Research
CTS: Cancer Thriving and Surviving
GEE: Generalized estimating eqs.
IT: Information technology
NCI: National Cancer Institute
NGS: Next generation sequencing
PFS: Progression-free survival
US: United States
VHAN: Vanderbilt Health Affiliated Network
VICC: Vanderbilt-Ingram Cancer Center
VUMC: Vanderbilt University Medical Center
